# Increasing taxonomic and functional characterization of host-microbiome interactions by DIA-PASEF metaproteomics

**DOI:** 10.3389/fmicb.2023.1258703

**Published:** 2023-10-16

**Authors:** David Gómez-Varela, Feng Xian, Sabrina Grundtner, Julia Regina Sondermann, Giacomo Carta, Manuela Schmidt

**Affiliations:** Systems Biology of Pain, Division of Pharmacology & Toxicology, Department of Pharmaceutical Sciences, Faculty of Life Sciences, University of Vienna, Vienna, Austria

**Keywords:** metaproteomics, data-independent acquisition, parallel accumulation-serial fragmentation, mouse gut microbiome, chronic neuropathic pain, host-microbiome interactions

## Abstract

**Introduction:**

Metaproteomics is a rapidly advancing field that offers unique insights into the taxonomic composition and the functional activity of microbial communities, and their effects on host physiology. Classically, data-dependent acquisition (DDA) mass spectrometry (MS) has been applied for peptide identification and quantification in metaproteomics. However, DDA-MS exhibits well-known limitations in terms of depth, sensitivity, and reproducibility. Consequently, methodological improvements are required to better characterize the protein landscape of microbiomes and their interactions with the host.

**Methods:**

We present an optimized proteomic workflow that utilizes the information captured by Parallel Accumulation-Serial Fragmentation (PASEF) MS for comprehensive metaproteomic studies in complex fecal samples of mice.

**Results and discussion:**

We show that implementing PASEF using a DDA acquisition scheme (DDA-PASEF) increased peptide quantification up to 5 times and reached higher accuracy and reproducibility compared to previously published classical DDA and data-independent acquisition (DIA) methods. Furthermore, we demonstrate that the combination of DIA, PASEF, and neuronal-network-based data analysis, was superior to DDA-PASEF in all mentioned parameters. Importantly, DIA-PASEF expanded the dynamic range towards low-abundant proteins and it doubled the quantification of proteins with unknown or uncharacterized functions. Compared to previous classical DDA metaproteomic studies, DIA-PASEF resulted in the quantification of up to 4 times more taxonomic units using 16 times less injected peptides and 4 times shorter chromatography gradients. Moreover, 131 additional functional pathways distributed across more and even uniquely identified taxa were profiled as revealed by a peptide-centric taxonomic-functional analysis. We tested our workflow on a validated preclinical mouse model of neuropathic pain to assess longitudinal changes in host-gut microbiome interactions associated with pain - an unexplored topic for metaproteomics. We uncovered the significant enrichment of two bacterial classes upon pain, and, in addition, the upregulation of metabolic activities previously linked to chronic pain as well as various hitherto unknown ones. Furthermore, our data revealed pain-associated dynamics of proteome complexes implicated in the crosstalk between the host immune system and the gut microbiome. In conclusion, the DIA-PASEF metaproteomic workflow presented here provides a stepping stone towards a deeper understanding of microbial ecosystems across the breadth of biomedical and biotechnological fields.

## Introduction

The characterization of microbial communities has increasingly gained attention due to their crucial role for the health across planetary ecosystems (Clemente et al., 2012; Prescott et al., 2018). However, our understanding of dynamic and multidimensional interactions between members of these ecosystems (hosts, bacteria, pathogens), and of the resulting functional alterations is still limited. Genomic sequencing methods are the most common approaches to studying these interactions by means of characterizing the taxonomic composition and relative abundance of functional genes. Nevertheless, the presence of a protein-coding gene does not always result in its expression under any given condition and even transcript levels can only partially predict protein levels (Liu et al., 2016). Thus, prediction algorithms are used for inferring the functional roles of microorganisms in an ecosystem based on genomic or transcriptomic datasets, yet such algorithms exhibit limited performance (Mills et al., 2019; Sun et al., 2020). Moreover, the inference on how changes in the abundance of specific taxa affect the functional output of a microbial habitat is unclear given the prominent functional redundancy (Fetzer et al., 2015; Tian et al., 2020). In fact, functional changes in the microbiome underlie gut inflammation and xenobiotic treatments without detectable changes in the taxonomic composition of the microbiome (Li et al.,^ 2023^). To overcome these boundaries, mass spectrometry-based proteomics applied to the study of hundreds of microbial organisms (termed, metaproteomics (Rodriguez-Valera, 2004; Wilmes and Bond, 2004)) has emerged as an attractive alternative. Metaproteomics has the potential to understand complex host-microbiome interactions as it enables the analysis of entire sets of proteins, providing direct insights into the identity and functionality of microorganisms present in an ecosystem (Stamboulian et al., 2022). However, proteomics acquisition methods classically used in metaproteomics have reached their theoretical profiling limits (Duan et al., 2022).

The predominant method employed for the acquisition of metaproteomic data is data-dependent acquisition (DDA) mass spectrometry (MS). DDA generally fragments only the most intense peptide ions, rendering the majority of remaining peptides unidentifiable (Michalski et al., 2011). Additionally, accurate quantification is challenging, owing to the inconsistent recording of ion intensities along the chromatographic profile. Consequently, reproducibility across repeated analyses is hampered and a sensitivity bias toward high-abundance peptides is introduced. These limitations are exacerbated in highly complex microbial samples (e.g., feces), which are estimated to be composed of 100 million peptide species (Armengaud, 2023) distributed across a very high dynamic range (Duan et al., 2022). To overcome these constraints, recent studies have evaluated the performance of data-independent acquisition (DIA) mass spectrometry (Aakko et al., 2020; Long et al., 2020; Zhao et al., 2022, 2023). Although DIA offers reproducibility improvements over DDA methods, spectral complexity and sampling efficiency remain challenging (Meier et al., 2020), particularly in highly complex peptidomes. Consequently, improved proteomic acquisition methods with the potential to increase the depth and resolution of metaproteomics are needed.

Previously, we showed how the combination of the Parallel Accumulation-Serial Fragmentation (PASEF) technology (Meier et al., 2015) and data analysis based on deep neuronal networks (Demichev et al., 2020), significantly increases the number of proteins quantified in mouse tissues (Xian et al., 2022). PASEF incorporates an additional ion mobility separation that allows for the differentiation of peptide signals that would otherwise be co-fragmented (Meier et al., 2015). This results in a more than tenfold increase in MS/MS scan rates without loss of sensitivity. Here, we aimed to investigate whether PASEF can optimize metaproteomics in complex microbial samples, i.e., in mouse feces.

In this study, we show that a DDA-PASEF-based workflow increased peptide identification up to 5 times and significantly improved data consistency and quantification reproducibility compared to previously published classical DDA (Kolmeder et al., 2016) and DIA (Aakko et al., 2020) methods. Moreover, we demonstrated that a DIA-PASEF strategy sets new profiling standards in metaproteomics by enabling the quantification of more proteins than DDA-PASEF while requiring only 1/10 of peptides. In comparison to previous DDA-based metaproteomic studies (Tanca et al., 2017, 2018), DIA-PASEF profiled up to 4 times more taxonomic units using 16 times fewer injected peptides and 4 times shorter chromatography gradients. Furthermore, DIA-PASEF offered comparable taxonomic depth as previous metagenomic studies. When applied to a pre-clinical mouse model of neuropathic pain, DIA-PASEF enabled the quantification of more than 15,000 protein groups and deciphered novel host-microbiome interactions associated with the establishment of chronic pain. Taken together, we expect DIA-PASEF to shed light on previously unexplored regions of the metaproteome and significantly enhance our understanding of microbiological ecosystems.

## Materials and methods

### Reagents

All reagents were purchased from Sigma-Aldrich (St. Louis, Missouri) if not mentioned otherwise. Acetonitrile (ACN) and formic acid (FA) were purchased from Fisher Scientific (Hampton, New Hampshire; both FA and ACN were liquid chromatography-mass spectrometry (LC–MS) grade). LC–MS grade water from Sigma was used for all solutions. Protease inhibitor was purchased from Roche (Complete Ultra Tablets Mini, Roche, Basel, Switzerland).

### Animal housing and surgery

In-house bred C57BL/6 J female mice were used. All animal experiments were carried out with the approval of the IACUC at the University of Vienna and of the Austrian Ministry for Education, Science and Research (BMBWF; license number 2021–0.138.925). All mice used in this study (four mice in each of the three experimental conditions) were group-housed in the same room, with a 12 h light/dark cycle, and with water and food *ad libitum*. All mice were weaned between 22–24 days after birth. The spared nerve injury (SNI) paradigm was used to induce neuropathic pain at 4 weeks of age. Mice were anaesthetized using isoflurane (4% for induction first and 2% for maintenance in O_2_). To expose the trifurcation of the left sciatic nerve, the skin and underlying biceps femoris muscle were incised. A ligature was placed around the common peroneal and tibial nerves with a 7–0 surgical braided silk (Vömel, Kronberg, Germany) below the bifurcation, followed by distal transection of both nerves. A 2 mm segment was excised from both nerves to impede regrowth. The skin was closed with one surgical clip (AutoClip®, Fine Science Tools, Heidelberg, Germany) and disinfected with povidone-iodine (Mundipharma, Frankfurt am Main, Germany). For analgesia mice were injected with carprofen (0.05 mL/10 g body-weight, Zoetis Österreich GmbH, Austria) immediately before surgery and on postoperative day 1. The clips were removed on postoperative day 5 with an AutoClip® remover (Mikron Precision Inc., Biel, Switzerland). In SHAM-operated mice, the same surgical procedure was carried out as in SNI but without ligating and transecting the branches of the sciatic nerve. All operated mice were weighed daily until postoperative day 7 and from then on, every second day until postoperative day 14 by female experimenters only (Sorge et al., 2014). Of note, the selection of which mice were used for SHAM or SNI surgeries was random and blinded to their microbiome profile, which was obtained only after the analysis of the fecal samples.

### Mechanical sensitivity test

The test was performed 3–4 days before surgery (Pre) and on a postoperative day 14 (14D) using a dynamic plantar aesthesiometer (automated von Frey filament, Dynamic Plantar Aesthesiometer: 37450–001, Dynamic Plantar Aesthesiometer Touch Stimulator: 37400–002, Ugo Basile®, Gemonio, Italy). Mechanical force (Force Intensity: 10.0 g, Ramp Time: 40s) was applied to the lateral side of the plantar hind paw and the withdrawal latency (in seconds) was measured five times for each hind paw, with at least a 2-min recovery period between each measurement. The test was done only by female experimenters (Sorge et al., 2014).

### Fecal collection

Mice were placed into 10x10cm boxes covered with a lid for 15 min. After removing mice from the boxes, feces were collected using forceps that were previously cleaned with 70% ethanol between each sample. Samples were transferred to precooled 2 mL autoclaved tubes (Eppendorf, Hamburg, Germany) and stayed on ice until stored at −80°C.

### Protein extraction, SP3-assisted protein digestion, and peptide clean-up

For protein preparation, 30 mg of fecal samples were mixed with 200 μL of lysis buffer (5% SDS, 2 M Urea, 50 mM Tris–HCl, Protease Inhibitor 1x) and vortexed vigorously for 1 min. Following, samples were placed in a Thermomixer (Serial Number: 5382JR638726, Eppendorf, Hamburg, Germany) for 15 min at 1,200 rpm and 70°C. When finished, samples were ultrasonicated using a Bioruptor Pico (Diagenode, Seraing, Belgium; program: 15 cycles of 30 s “ON” and 30 s “OFF,” frequency level: Low, water temperature: +20°C). Afterwards, tubes were centrifuged at 16,000 g for 5 min at room temperature. Supernatants were saved in new tubes and submitted to a new round of centrifugation. The saved supernatants were stored at −80°C until SP3-assisted protein digestion.

For protein clean-up and digestion, a modified version of the single-pot, solid-phase-enhanced sample preparation (SP3) method from Hughes et al. was used (Hughes et al., 2019). Briefly, 50 μg protein was taken into a 1.5 mL LoBind tube (Eppendorf, Hamburg, Germany), and the sample volume was added up to 50 μL with lysis buffer. The fecal sample was subjected to protein reduction (5 mM Dithiothreitol, DTT, 30 min incubation at +60°C) and alkylation (20 mM Iodoacetamide, IAA, 30 min at room temperature in the dark). The remaining IAA in the sample was quenched with the addition of DTT to a final concentration of 5 mM. 10 μL of pre-mixed Sera-Mag SpeedBead beads (50 mg/mL, Cytiva, Marlborough, Massachusetts) were added to 50 μg protein sample. To initiate the binding of proteins to the beads, one volume of absolute ethanol was added immediately, followed by incubation on a Thermomixer (Eppendorf) at 24°C for 5 min with 1,000 rpm agitation. The supernatant was removed after 2 min resting on a magnetic rack, and the beads were rinsed three times with 500 μL of 80% ethanol. Rinsed beads were reconstituted in 50 μL digestion buffer (50 mM ammonium bicarbonate, pH 8). Protein digestion was performed with 2 μg of sequencing-grade trypsin/LysC (Promega, Madison, United States) for 18 h at 37°C with 950 rpm agitation. After digestion, ACN was added to each sample to a final concentration of 95%. Mixtures were incubated for 8 min at room temperature and then placed on a magnetic rack for 2 min. The supernatant was discarded, and the beads were rinsed with 900 μL of 100% ACN. Rinsed beads were reconstituted in 20 μL LC–MS grade water to elute the peptides. The peptide concentration was measured in duplicate using NanoPhotometer N60 (Serial number: TG2022, Implen, Munich, Germany) at 205 nm. Peptide samples were acidified with FA to a final concentration of 0.1% and stored at −20°C until LC–MS/MS analysis.

### LC–MS/MS setup

Nanoflow reversed-phase liquid chromatography (Nano-RPLC) was performed on a NanoElute system (Bruker Daltonik, Bremen, Germany). Peptides were separated with either a 70 min or 130 min gradient on a 25 cm x 75 μm column packed with 1.6 μm C18 particles (IonOpticks, Fitzroy, Australia). Mobile solvent A consisted of 2% ACN, 98% water, 0.1% FA and mobile phase B of 100% ACN + 0.1% FA. For both gradient lengths, the flow rate was set to 400 nL/min for the first 2 min and the last 9 min of the gradient, while the rest of the gradient was set to 250 nL/min. In the 70 min separation, the mobile phase B was linearly increased from 0 to 20% from 3 min to 50 min, followed by a linear increase to 35% within 10 min and a steep increase to 85% in 0.5 min. Then a flow rate of 400 nL/min at 85% was maintained for 9 min to elute all hydrophobic peptides. In the 130 min separation, the mobile phase B was linearly increased from 0 to 20% from 3 min to 110 min, followed by a linear increase to 35% within 10 min and a steep increase to 85% in 0.5 min. Then a flow rate of 400 nL/min at 85% was maintained for 9 min to elute all hydrophobic peptides. NanoElute LC was coupled with a hybrid TIMS quadrupole TOF mass spectrometer (timsTOF Pro, Bruker Daltonik, Bremen, Germany) *via* a CaptiveSpray ion source. Samples were analyzed in both data-independent acquisition (DIA) and data-dependent acquisition (DDA) modes coupled with parallel accumulation serial fragmentation (PASEF) for methods comparison. The samples used for the neuropathic pain characterization (SNI, SHAM and Naive) were analyzed in DIA-PASEF. In both acquisition modes, the TIMS analyzer was operated in a 100% duty cycle with equal accumulation and ramp times of 100 ms each. Specifically, in DDA-PASEF mode (Meier et al., 2018), 10 PASEF scans were set per acquisition cycle with ion mobility range (1/k0) from 0.6 to 1.6, and singly charged precursors were excluded. Dynamic exclusion was applied to precursors that reached a target intensity of 17,500 for 0.4 min. Ions with m/z between 100 and 1700 were recorded in the mass spectrum. In DIA-PASEF mode (Meier et al., 2020), precursors with m/z between 400 and 1,250 were defined in 16 scans containing 32 ion mobility steps with an isolation window of 26 Th in each step with 1 Da overlapping for neighbouring windows. The acquisition time of each DIA-PASEF scan was set to 100 ms, which led to a total cycle time of around 1.8 s. In both DDA- and DIA-PASEF modes, the collision energy was ramped linearly from 59 eV at 1/k0 = 1.6 to 20 eV at 1/k0 = 0.6.

### Protein database generation for metaproteome analysis

A metagenome-translated protein database (PD1) was downloaded from http://gigadb.org/ containing 2.6 million protein sequences (Xiao et al., 2015). Due to the large size of the protein database, a two-step approach (Jagtap et al., 2013) was applied to generate a reduced and sample-specific protein database. Briefly, a fecal pooled peptide sample was recorded 10 times using DDA-PASEF and a 70 min chromatography gradient. The 10 raw data files were first converted into mgf format and then searched against the PD1 and a decoy database generated with reversed sequences using X!Tandem (Muth et al., 2010), a search engine integrated into SearchGUI (Barsnes and Vaudel, 2018) (Version 4.1.11). Trypsin was specified with a maximum of 2 missed cleavages allowed. The search included variable modifications of methionine oxidation and N-terminal acetylation and a fixed modification of carbamidomethyl on cysteine. The mass tolerances of 10 ppm for both precursor and fragment were used. The output of the X!Tandem search was further validated in PeptideShaker with 1% FDR at PSM, peptide and protein levels (Vaudel et al., 2015). All validated proteins were exported as the reduced protein database (PD2) containing 9,750 protein sequences. PD2 was further used for the comparison of different workflows. Another pooled peptide sample made from Naive, SHAM and SNI mice was submitted to a 130 min gradient and analyzed in DDA-PASEF mode in 9 replicates. The 9 raw data files were subjected to the same aforementioned procedures to generate another reduced protein database (PD3) containing 10,859 protein sequences. PD3 was further used to analyze the metaproteome of Naive, SHAM and SNI mice. *Mus musculus* reference proteome (PD4) was downloaded from Uniprot^1^ and used to identify host proteins from the fecal samples. To avoid misassignment of host- and microbiome-derived peptides, we used either PD2 or PD3 in combination with PD4 during the comparison of the different workflows and the analysis of neuropathic pain samples, respectively. To accurately compare the differences in performance across different acquisition modes (DDA- and DIA-PASEF) and the different data analysis solutions (MQ and DIA-NN), we used the same pooled fecal sample for all experiments shown in all figures except the last one. The use of this pooled sample prevents sample bias and ensures a generalized sample-independent representation of the mouse fecal microbiome.

### Data processing using different workflows

DDA-PASEF raw data files were analyzed with MaxQuant (version 2.1.3.0) and searched with Andromeda against PD2 and PD4, at the same time. The search type was specified as “TIMS-DDA.” The minimal peptide length was set to 7 amino acids, and a maximum of 2 missed cleavages was allowed.

The search included methionine oxidation, asparagine and glutamine deamidation and N-terminal acetylation as variable modifications, as well as cysteine carbamidomethylation as fixed modification; a maximum of 5 modifications per peptide was allowed. The “Match between runs” function was checked within a 0.7 min retention time window and 0.05 ion mobility window. Mass tolerance for peptide precursor and fragments were set as 10 ppm and 20 ppm, respectively. The FDR was set to 0.01 at the precursor level and protein level. Label-free quantification algorithm with a minimum 1 LFQ ratio count was used to quantify identified proteins. The rest of the parameters were kept as default.

DIA-PASEF raw data files were analyzed with MaxQuant (version 2.1.3.0) and the search type was specified as “TIMS MaxDIA.” The data were searched against the spectral library generated with the DDA-PASEF data search in the MaxQuant workflow mentioned above using the Andromeda algorithm. Specifically, the output evidence.txt, peptide.txt and msms.txt files were used. The rest of the configurations were set as same as the search for DDA-PASEF data.

DIA-PASEF raw data files were also searched in DIA-NN (Demichev et al., 2020). DIA-NN (version 1.8.1) was used to process DIA-PASEF data in library-free mode with PD2 or PD3 and PD4 to generate the predicted spectrum library. A deep learning-based method was used to predict theoretical peptide spectra along with their retention time and ion mobility. Trypsin/P was used for *in silico* digestion with an allowance of a maximum of 2 missed cleavages. Variable modifications on peptides were set to methionine oxidation and N-term acetylation, while N-term methionine excision and carbamidomethylation on cysteine were fixed modifications. The maximum number of variable modifications on a peptide was set to 5. Peptide length for the search ranged from 7 to 52 amino acids. Aligned with the DIA-PASEF acquisition method, m/z ranges were specified as 400 to 1,250 for precursors and 100 to 1700 for fragment ions. Both MS1 and MS2 mass accuracy were set to automatic determination. Protein inference was set to “Protein names (from FASTA)” and the option of “Heuristic protein inference” was unchecked. Match-between-run (MBR) was checked for cross-run analysis in all analyses performed in this study. RT-dependent cross-run normalization and Robust LC (high precision) options were selected for quantification.

In addition, the same DIA-PASEF raw data files were processed with DIA-NN (version 1.8.1) using an experimental spectrum library generated in our lab with several metaproteome studies. The experimental library contains microbial and host proteins of in total 14,711 protein isoforms, 19,153 protein groups and 98,159 precursors. Besides the use of the experimental spectrum library, all other search parameters were the same as described above for the DIA-PASEF data search in library-free mode.

### Metaproteome data batch correction and normalization

For the neuropathic pain experiment, precursor intensities (Normalized Intensity from the DIA-NN main report table) were submitted to median normalization and quantile batch correction using the proBatch R package (Cuklina et al., 2021). The resulting precursor intensities were further processed with the R package, DIA-NN,^2^ to extract and calculate the MaxLFQ (Cox et al., 2014) quantitative intensity for all identified peptides and protein groups with q-value <0.01 as criteria at precursor and protein group levels.

### Taxonomy and function annotation

iMetaLab (Cheng et al., 2020) (version 2.3.0) was used for taxonomy and function annotation. The MaxLFQ peptide data (microbial and host) were imported into iMetaLab, and the built-in taxonomy database was used for the mapping (Ignore blanks below rank: Superkingdom, Unique peptide count ≥3). MaxLFQ protein identifications with corresponding intensities were imported into iMetaLab for functional annotation (using default parameters). The K numbers (KEGG Orthology identifiers) of mapped proteins were extracted and analyzed online^3^ to obtain KEGG pathways and their corresponding number of mapped targets.

The analysis of the taxon-specific functions was performed using Meta4P (Porcheddu et al., 2023). The quantitative table of microbial peptides (i.e., Supplementary Table 2) and the taxonomic annotation table (i.e., Supplementary Table 3) were imported into Meta4P. Proteins annotated with different function databases were generated *via* eggNOG^4^ using PD2. Taxa-specific KEGG pathways were exported and a total number of at least 3 peptides per taxon-KEGG pathway was set as the threshold to filter the results.

### Taxonomic quantification strategy

The iMetalab’s taxonomy annotation output underwent further processing in R, where the following steps were taken to quantify the detected taxa in each sample. Firstly, peptides without annotations at a specific taxon level (e.g., Species) were removed. Next, taxa with at least three unique peptides annotated were retained. The peptide intensity in each sample was then extracted based on the retained taxa. Peptides that were not quantified in all samples were removed. Finally, the log2-transformed intensities of common peptides annotated in the same taxon were summed up in each sample. The intensities matrices of 14D and Pre in each condition were subjected to paired t-test in GraphPad Prism (version: 9.5.0(730)) using the “Benjamini and Hochberg” method for multiple comparisons.

### Functional quantification strategy

The output of the function annotation in iMetaLab underwent further processing in R using the following steps to calculate the intensity of each KEGG pathway in each sample using proteins with a significance *p* < 0.005 when comparing 14D versus Pre within each condition (paired *t*-test in Limma package (Ritchie et al., 2015)). Firstly, significantly regulated proteins mapped with KEGG ortholog numbers (K numbers) were kept for the following analysis. Secondly, different pathways annotated with the same K number were separated into rows with duplicated intensities for this entry in all samples, as there is no evidence to suggest that the protein belongs to only one pathway. Thirdly, the intensities of annotated numbers mapped to the same pathway were summed up in each sample. Fourthly, the number of mapped K numbers to each pathway in each sample was counted. Finally, the intensity of each pathway was normalized by dividing the summed intensity by the total K numbers mapped for each pathway in each sample. The intensity of each pathway was log2-transformed before statistical analysis. The transformed intensities were tested by comparing 14D versus Pre in each condition (Naive, SHAM, and SNI) using the Limma (Ritchie et al., 2015) R package in a paired manner with the “Benjamini and Hochberg” method for multiple comparisons.

### Pathway analysis of host proteome

The host protein identifications were analyzed in Metascape (Zhou et al., 2019) (https://metascape.org/, version 3.5.20230101, accessed on 2023.02.17) and searched against the Gene Ontology Molecular Function database with default settings.

## Results

### Development of a PASEF-based metaproteomic workflow

**Figure 1:**
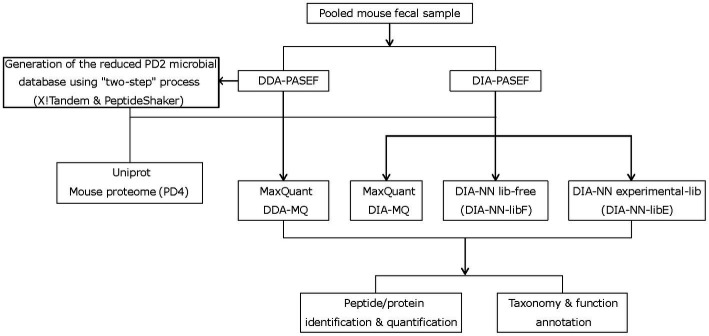
Study workflow depicting the two-step strategy with X!Tandem and PeptideShaker, different mass-spectrometry acquisition modes, and data processing steps.

Current metaproteomic methods have reached their identification and quantification limits in complex microbial samples with high dynamic range (Duan et al., 2022) (e.g., feces). We speculated that the extra layers of information accessible by the PASEF technology (Meier et al., 2015, 2021) could offer significant improvements similar to what we demonstrated in our recent work on complex mouse tissue samples (Xian et al., 2022). PASEF creates an extra ion mobility dimension that increases the sequencing speed ten-fold and reduces spectral complexity. To this end, mouse peptide fecal samples were analyzed on a timsTOF Pro mass spectrometer (Bruker Daltonics) equipped with a dual TIMS cartridge and using the PASEF acquisition method (Meier et al., 2018) (Figure 1). In addition to implementing PASEF using a DDA acquisition scheme (DDA-PASEF), we also tested the potential advantages of the DIA-PASEF acquisition mode (Meier et al., 2020), which produces nearly complete datasets with peptide features defined in a four-dimensional data space (retention time, m/z, ion mobility, and intensity). Finally, we benchmarked two of the most commonly used publicly available software solutions for analyzing multi-dimensional datasets generated in DDA- and DIA-PASEF modes, MaxQuant (Cox and Mann, 2008) and DIA-NN (Demichev et al., 2022).

### Identification and quantification performance

To compare the performance of different PASEF acquisition modes and data analysis software solutions, we first evaluated the total amount of microbial and mouse peptides and proteins, which were quantified in 10 technical replicates of a pooled mouse fecal sample (500 ng of total peptides per run). The data generated in DDA-PASEF mode were analyzed within MaxQuant (DDA-MQ), while DIA-PASEF data were analyzed using the TIMS MaxDIA module of MaxQuant (DIA-MQ) and DIA-NN. For the latter, we compared the performance employing an experimentally generated spectral library (DIA-NN-libE) to the library-free mode (DIA-NN-libF). In all cases, we set a protein and precursor FDR cutoff of 1%, as calculated by each software.

**Figure 2:**
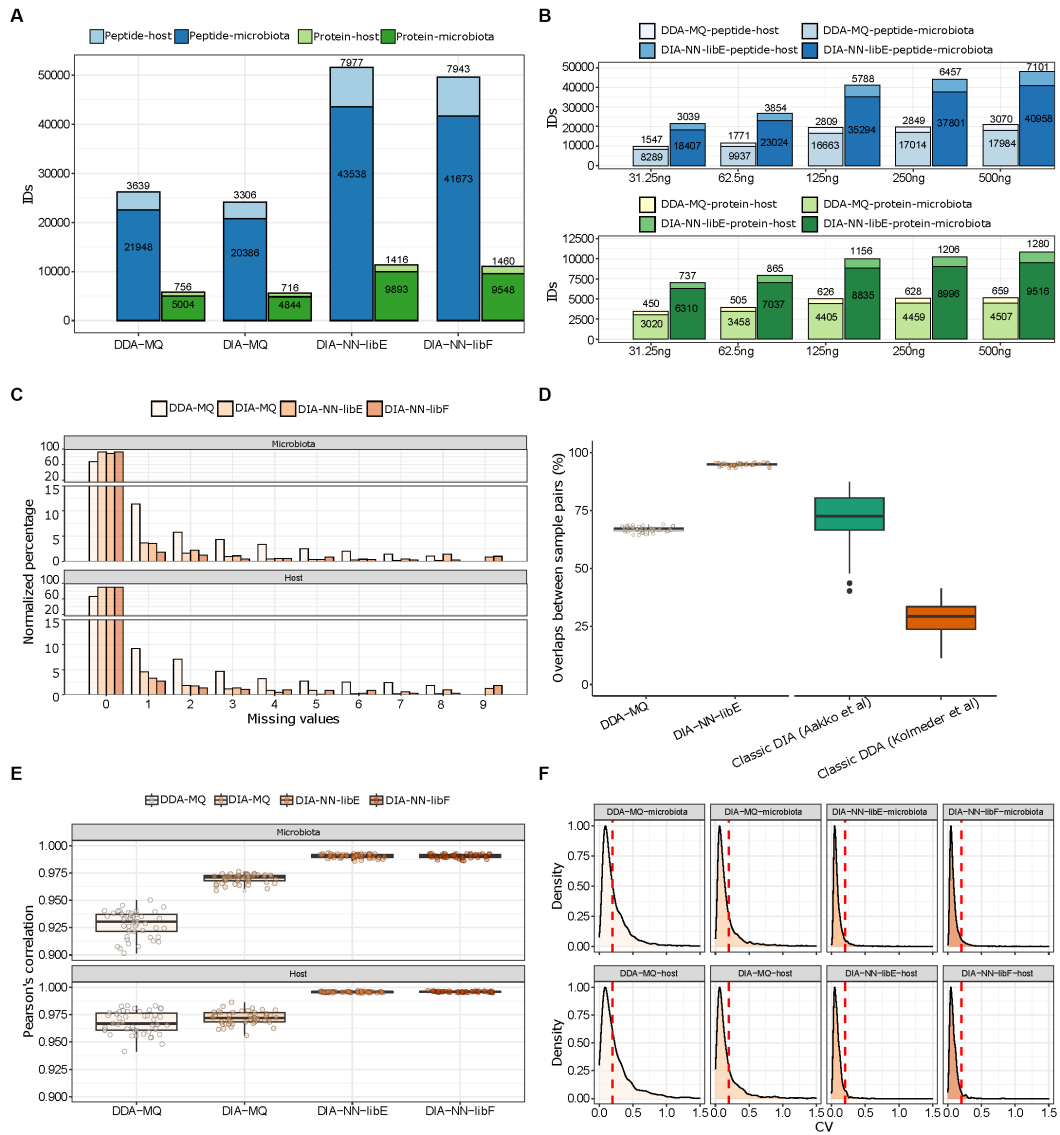
Performance evaluation of DDA- and DIA-PASEF analyzed with indicated software solutions. **(A)** Total amount of microbial and host peptides (dark and light blue, respectively) and proteins (dark and light green, respectively) identified in 10 technical replicates of a pooled mouse fecal sample in four different workflows. **(B)** Number of peptide (blue colors) and protein (green colors) identifications in either DDA-MQ or DIA-NN-libE workflows at different peptide injection quantities analyzed in triplicates. **(C)** Data completeness of microbial (upper) and host (lower) protein identifications in different workflows across 10 technical replicates. **(D)** Overlap of peptide identifications across all paired comparisons from 10 technical replicates in DDA-MQ and DIA-NN-libE workflows, and comparison with 2 previous studies. **(E)** Intra-group correlations of 4 workflows across all paired comparisons from 10 technical replicates. The dots indicate the Pearson’s correlation for each possible paired comparison of proteins. **(F)** Coefficient of variation (CV) distribution of proteins in the microbiota (upper) and host (lower) across 10 technical replicates in 4 workflows. The red dotted line indicates the CV = 0.2.

We found that DIA-NN-libE yielded nearly two-fold improvements in the number of identified microbial peptides and proteins compared to DDA-MQ (Figure 2A). Due to the depth of the spectral library generated by DDA-MQ, DIA-MQ identifications were clearly limited (Figure 2A). In addition, the amount of identified host peptides and proteins were also increased two-fold (Figure 2A; Supplementary Table 1). Similar results were obtained when using the library-free mode (DIA-NN-libF; Figure 2A). This performance boost resulted in a fifteen-fold increase in sensitivity as highlighted by a higher detection of microbial and host peptides and proteins by DIA-NN-libE using 31.25 ng of peptides compared to 500 ng analyzed by DDA-MQ (Figure 2B; Supplementary Table 2). Importantly, all tested DIA approaches generated data matrices with considerably fewer missing identifications for both the microbial and host proteins, compared to DDA (Figure 2C). A comparison with representative studies using a classical DIA (Aakko et al., 2020) and a classical DDA methods (Kolmeder et al., 2016) revealed that the peptide overlap enabled by the DDA-PASEF vastly improved compared to classical DDA and reached similar performance as previously published classical DIA strategies. Further, DIA-NN-libE set new standards of reproducibility compared to the aforementioned methods (Figure 2D). This optimization of identification performance was not due to differences in FDR algorithms used by each software, as shown by a two-species strategy (Supplementary Figure 1). On the contrary, DIA-NN-libF detected significantly fewer false positive precursors than DDA-MQ at any given FDR level (Supplementary Figure 1).

We further critically evaluated the quantification performance of the DDA- and DIA-PASEF approaches. DIA-NN-libE quantified 12,700–28,487 peptides not detected by DDA-MQ at any injected amounts tested (Supplementary Figure 2A). Importantly, a detailed analysis showed that the quality score distribution of the peptides was similar between the ones uniquely identified by DIA-PASEF and the ones commonly identified by DDA- and DIA-PASEF (Supplementary Figure 2B). Notably, DIA-PASEF detected peptides covering over 4 orders of abundance magnitude, displaying the biggest gains, compared to DDA-PASEF, in the lower intensity ranges (Supplementary Figure 2C). These improvements were accompanied by better protein intensity correlations among all ten technical replicates in all three DIA-PASEF-based analyses (Figure 2E), which was preserved even at the lowest sample amount tested (Supplementary Figure 3). As a result of this superior quantification accuracy, the number of proteins with a coefficient of variability (CV) < 20% significantly increased in DIA-PASEF (61% in DDA-MQ, 75% in DIA-MQ, and 94.49% in DIA-NN-libE; Figure 2F). In summary, our data clearly show the benefits of the fourth data dimension unlocked by PASEF highlighting the potential of DIA-PASEF for serving as a new state-of-the-art acquisition method in metaproteomics.

### Taxonomic and functional profiling

**Figure 3:**
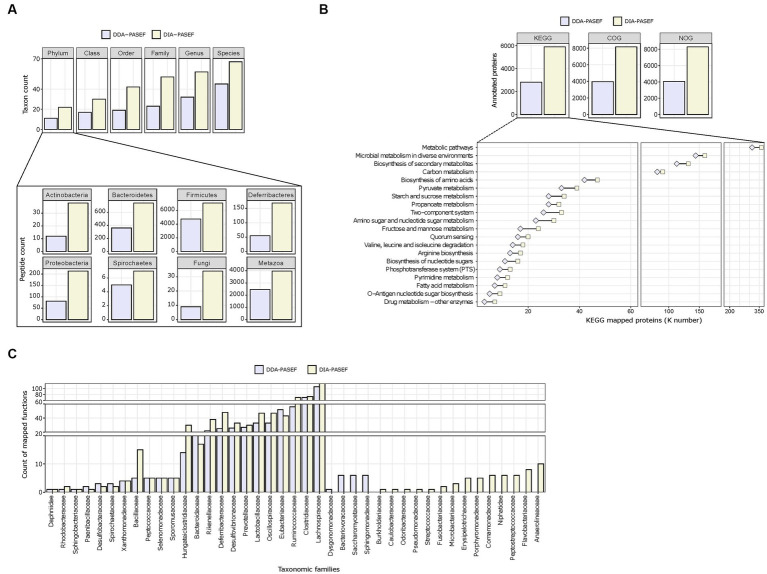
Taxonomic profiling and functional annotations of metaproteomes. We used identified peptides and protein groups from 3 technical replicates (250 ng peptide per MS run) in DDA-MQ or DIA-NN-libE workflows. **(A)** Comparison of DDA-PASEF (lavender) and DIA-PASEF (beige) workflows at different taxonomic levels (upper panel: cut-off ≥3 unique peptides per taxon as identified in iMetalab). The zoom-in bar plots show phyla annotated in both workflows with their respective peptide counts. **(B)** Bar plots show the number of microbial proteins annotated using KEGG, COG, and NOG databases. The zoom-in lower panel depicts selected KEGG pathways (details in Supplementary Table S5) with the number of microbial proteins mapped to KEGG ortholog identifiers (K numbers). **(C)** A given example of taxon-function analysis at the family level in comparison to DDA- and DIA-PASEF data using Meta4P.

We next assessed the performance of DDA-PASEF and DIA-PASEF in identifying microbial taxa. Globally, DIA-PASEF identified organisms belonging to the four major kingdoms present in the mouse gut microbiome (Supplementary Figure 4A). Using a cut-off of at least three taxon-specific peptides, DIA-PASEF increased the identification performance from 1.48-fold (at the species level) to 2.26-folds (at the family level) compared to DDA-PASEF (Figure 3A; Supplementary Table 3). A detailed view revealed remarkable identification gains for several phyla such as *Actinobacteria*, *Bacteroidetes*, and *Firmicutes* in addition to fungi and metazoans (Figure 3A). DIA-PASEF detected more genera known to constitute the core gut microbiome of healthy mice reaching a lower limit of 0.04% relative abundance, as established by 16S rRNA sequencing (Wang et al., 2019) (genus: *Gordonia*; Supplementary Table 4). Moreover, the peptide coverage was significantly increased for 34 of the 35 genera commonly detected by DIA- and DDA-PASEF (Supplementary Table 4).

Next, we investigated the annotation of detected proteins to functional pathways for both the microbiota and the host. The aforementioned improvements in protein quantification reached by DIA-PASEF translated to increased numbers of KEGG, COG, and NOG functional terms compared to DDA-PASEF (Figure 3B). A detailed analysis showed that DIA-PASEF increased the coverage of protein groups with general or unknown functions: DIA-PASEF enabled the quantification of 485 protein groups annotated with COG category R (General function prediction only) and category S (unknown function), in comparison to 220 protein groups annotated in these two categories with DDA-PASEF (Supplementary Table 3). DIA-PASEF data covered 100% of all KEGG pathways detected by DDA-PASEF (Figure 3B) and enabled the quantification of additional 11 pathways (Supplementary Table 5). Moreover, DIA-PASEF offered higher coverage in nearly 50% of commonly annotated pathways (Figure 3B and Supplementary Table 5).

Of particular interest is the fact that DIA-PASEF increased the detection of functions assigned to specific taxonomic families (Figure 3C). The number of KEGG entries assigned by peptides detected using DIA-PASEF increased for the majority of members at all taxonomic ranks (family level in Figure 3C, all other ranks in Supplementary Table 6), which highlights the benefits of the workflow.

As for the host biology, DIA-PASEF enabled the characterization of 77 unique protein functional categories (Supplementary Table 5), as well as an increased number of host proteins associated with Gene Ontology Molecular function terms (GO-MF) commonly detected by DDA-PASEF (Supplementary Figure 4B and Supplementary Table 5).

### DIA-PASEF offers novel insights into host-microbiome interactions upon neuropathic pain

Chronic pain affects an estimated 20% of the world’s population (Treede et al., 2015; Price and Gold, 2018). Nevertheless, current therapies are ineffective for many patients and exhibit significant side effects (Price and Gold, 2018). Initial studies utilizing 16S rRNA sequencing showed changes in the gut microbiome composition during the onset and regulation of chronic pain in humans and animal models (Morreale et al., 2022), suggesting the gut microbiome as a potential target for novel treatments. However, our knowledge is scarce and a thorough assessment of functional alterations of the gut microbiome associated with chronic pain is awaiting.

**Figure 4:**
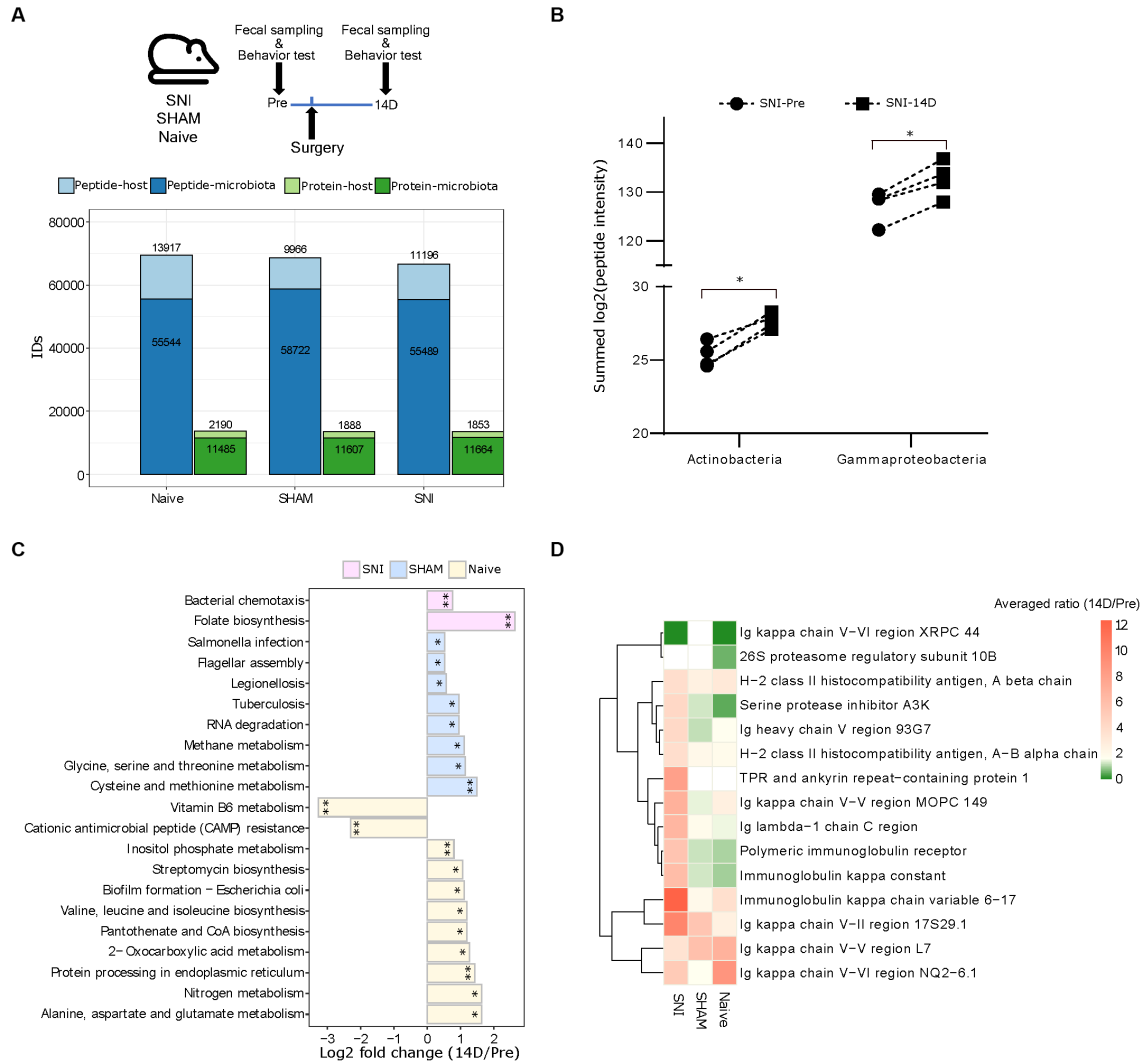
Host-microbiome changes in the mouse gut in a preclinical mouse model of neurophatic pain (SNI). **(A)** *Top*: Experimental workflow indicating the three experimental groups and the times of fecal sampling, surgery (i.e., nerve-injury), and behavioural assessment (for details, please see Methods). *Bottom*: Peptide and protein identifications in both the microbiota and the host using DIA-PASEF in all three experimental groups (Naive, SHAM, and SNI). **(B)** The abundance of the two indicated bacterial classes that are specifically up-regulated in SNI mice at 14D post-surgery (^*^q < 0.05; paired t-test with Benjamini-Hochberg correction for multiple comparisons). **(C)** Significantly altered KEGG pathways of the microbiome in all three experimental groups (^*^q < 0.05, ^**^q < 0.01). **(D)** Expression patterns of 15 selected host proteins (selected based on *p* < 0.005, pair-wise comparison 14D versus Pre in SNI) in all three experimental conditions.

We set out to test our DIA-PASEF metaproteomic workflow in the validated spared nerve injury (SNI) mouse model of neuropathic pain (Decosterd and Woolf, 2000). We collected fecal samples from four aged-matched SNI, four SHAM (surgery without nerve injury), and four Naive female mice before (Pre) and 14 days (14D) post-surgery covering the critical period of chronic pain onset (Cobos et al., 2018; Segelcke et al., 2021) (Figure 4A). Analysis of mouse behaviours confirmed the expected development of neuropathic pain as indicated by mechanical allodynia (Cobos et al., 2018) (i.e., hypersensitivity to an innocuous tactile stimulus to the affected hind paw) in SNI but not in SHAM mice (Supplementary Figure 5). In total, our DIA-PASEF workflow detected 81,378 peptides corresponding to 12,239 and 2,267 microbial and mouse protein groups, respectively (Figure 4A; Supplementary Table 7). We quantified 29 phyla, 45 classes, and 119 species with at least 3 peptides per taxon (Supplementary Figure 6A and Supplementary Table 8). A comparison to two previously published classical DDA mouse gut metaproteomic studies (utilizing 16 times more injected peptides, and 4 times longer chromatography gradients per run (Tanca et al., 2017, 2018)), showed the ability of our DIA-PASEF workflow to profile up to 4 times more taxonomic units (Supplementary Figure 6B). Additionally, we harnessed a recent compilation of 2,446 global mouse gut metagenomes (Beresford-Jones et al., 2022) as a reference for microbiota abundance. Remarkably, DIA-PASEF exhibited high sensitivity and detected species with a mean abundance of 0.003 (when using the cutoff of 3 species-specific peptides, i.e., *Alistipes indistinctus*; Supplementary Table 4).

From the taxonomic point of view, we revealed that the bacterial classes *Actinobacteria* and *Gammaproteobacteria* were increased during neuropathic pain in SNI animals at 14D (Figure 4B; Supplementary Table 9). Besides taxonomy, metaproteomics facilitates insights into host-microbiome interactions. To reveal functional changes in host-microbiome interactions upon nerve injury, we investigated proteins and their association with biological pathways. Pathway analysis using KEGG terms showed significant enrichment of distinct microbial pathways in each of the three experimental conditions when comparing 14D with “Pre” data (Figure 4C; Supplementary Table 9). With respect to the functional changes in the gut proteome of the host (i.e., in mice), there was only limited overlap across all three conditions; instead, we observed rather quite specific pathway alterations (Supplementary Figure 7A): while SNI animals exhibited upregulation of processes related to the immune response of the gut (Figure 4D), in SHAM animals the repertoire of altered proteins was distinct, and the extent of observed changes was lower except for the increase of several major urinary proteins (Supplementary Figure 7B). Notably, unsupervised clustering of protein intensities “Pre” surgery strongly suggests that observed condition-specific alterations were not due to baseline gut proteome differences between the mice (Supplementary Figure 7C; importantly, the mice were randomly assigned to the three experimental conditions before the surgeries, please see Methods for details). Taken together, our results provide a stepping stone for follow-up interventional studies investigating the mechanistic relevance of here discovered host-microbiome alterations for neuropathic pain.

## Discussion

We present a novel metaproteomic workflow utilizing PASEF technology, as well as deep neural network-based data analysis offered by DIA-NN. The combination of both elements yielded very significant improvements compared to previous metaproteomic workflows and facilitated, for the first time, the global assessment of host-microbiome interactions in a mouse model of neuropathic pain.

We hypothesized that the features of the PASEF technology would be especially advantageous in a sample with high dimensionality like the fecal microbiome, where few species represent more than 80% of the total abundance (Duan et al., 2022). The sequential release of ion packages trapped in the tunnel of the TIMS mobility analyzer together with the synchronization of the quadruple selection increases sensitivity and reduces the complexity of spectra generated by PASEF (Meier et al., 2015). Accordingly, we show that the reproducibility of detection between samples offered by DDA-PASEF doubled compared to classical DDA approaches and even matched the performance of classical DIA (Aakko et al., 2020). Adding a DIA mode to the PASEF scheme enabled us to even further increase the number of peptides and proteins quantified in comparison to DDA-PASEF. These gains became overtly evident in peptides at the lower abundance range, a fact that will greatly improve the detection limits of current metaproteomic workflows (Duan et al., 2022). The CScore distribution of the peptides was similar between the ones uniquely identified by DIA-PASEF and the commonly identified by DDA- and DIA-PASEF, which establishes high confidence in identification gains enabled by DIA-PASEF. Notably, DIA-PASEF reached similar or even better taxonomic depth than previous large-scale mouse metagenomic studies (using 16S rRNA). Upcoming DIA-PASEF schemes (Szyrwiel et al., 2022; Distler et al., 2023; Skowronek et al., 2023) as well as other DIA data analysis tools (Zhang et al., 2023) have the potential to further advance the impact of PASEF on metaproteomics that we pioneered in this study.

DIA-PASEF achieves a nearly complete record of all peptide fragments (Meier et al., 2020). Despite the significant improvements facilitated by DIA-PASEF, only 31,957 out of 66,748 microbial peptides (47%) identified during the analysis of the neuropathic pain samples were taxonomically annotated highlighting a limitation in the depth of currently available taxonomic databases. In addition, a large amount of peptide spectra likely remains unidentified. Nonetheless, this information is stored in very comprehensive digital MS/MS datasets. Thus, our workflow offers the possibility to interrogate these data in the future by either using continuously improved microbiome-based databases or emerging AI-based algorithms. The use of these datasets in the metaproteomic field will offer extra advantages: community-shared data resources, optimization in the utilization of valuable microbiome samples (e.g., clinical human samples), and reduced animal experimentation for hypothesis testing.

The taxonomic and functional depths achieved by DIA-PASEF allowed us to directly interrogate, for the first time, the functional alterations in the gut ecosystem during neuropathic pain. Our findings suggest that *Actinobacteria* and *Gammaproteobacteria* are increased upon neuropathic pain in SNI animals. These data are in line with previous genomic findings in SNI mice (Hua et al., 2021) and interstitial cystitis human pain syndrome (Braundmeier-Fleming et al., 2016).

Far beyond the extent of previous taxonomic studies, we detected an upregulation of the microbial KEGG pathway “folate biosynthesis” exclusively upon nerve injury. Folate is produced in the murine gut by several bacterial taxa including *Actinobacteria* (Engevik et al., 2019), and its deficiency has been associated with a greater risk of peripheral neuropathy in a retrospective cohort of more than 500,000 people (Taverner et al., 2019). Remarkably, Botez et al. (1978) showed an improvement in clinical and electrophysiological measurements of five patients with polyneuropathy after 9–39 months of folate therapy. Therefore, the functional insights offered here might point towards an endogenous mechanism, by which the gut ecosystem aims at ameliorating neuropathic pain. This is an exciting hypothesis that may open new therapeutical avenues harnessing folate supplementation as already implemented for diverse autoimmune diseases (Mölzer et al., 2021). Folate was reported to stabilize and increases the abundance of T_reg_ lymphocytes in the colon (Mölzer et al., 2021). Interestingly, T_reg_ lymphocytes can alleviate chronic neuropathic pain by inhibiting the Th1 response *via* CD4+ helper cells (Bethea and Fischer, 2021). Emerging clinical evidence proposes an intricate interplay between the gut microbiome, neuroimmune signalling and neuropathic pain (Ustianowska et al., 2022), e.g., by influencing the balance between pro-inflammatory and anti-inflammatory T cells (Ding et al., 2021).

In addition to the potential role of bacterial folate production, we also discovered alterations of several host protein complexes, exclusively in SNI animals. For example, those known to be involved in the crosstalk between the host immune system and the gut microbiome. We observed an upregulation of two components of the Major Histocompatibility Complex II (MHC-II). MHC-II contributes to the maturation of B cells *via* the presentation of exogenous bacterial antigens and the production of secreted IgA (SIgA) (Jiang et al., 2019). Dysregulation of the IgA-microbiota axis affects multiple pathologies with an inflammatory component (Abokor et al., 2021), but its link to pain syndromes is unknown. Notably, we observed a significant upregulation of the Polymeric Immunoglobulin Receptor in SNI animals at 14D. Polymeric Immunoglobulin Receptor represents one of the two proteins that are necessary for the secretion of IgA from gut mucosal plasma cells into the gut lumen where it contributes to controlling the abundance of commensal microbiota (Weis and Round, 2021). A detailed analysis of our data shows that both the immunoglobulin J chain (J chain), also being part of this secretory complex, as well as IgA tend to be upregulated in SNI animals, even though they did not pass our statistical cutoff (Uniprot IDs: P01592 and P01878 in Supplementary Table 10). Furthermore, we observed a significant increase in *Gammaproteobacteria* in SNI mice. In this context, it is noteworthy that *Gammaproteobacteria* are known to produce gut inflammation in mice thereby triggering an IgA-dependent immune response *via* activation of B cells (Mirpuri et al., 2014). Thus, our findings open new avenues for future mechanistic studies aimed at deciphering the role of these microbiome-immune pathways for chronic pain. Besides, our data revealed prominent functional changes occurring in the gut of age-matched Naive mice over the two-week experimental period, which highlights the need to adequately control for purely age-induced changes in microbiome studies by including Naive non-treated mice.

In summary, we present the valuable potential of DIA-PASEF to provide in-depth and novel insights into host-microbiome interactions with a significant impact on our understanding of microbiological ecosystems across diverse biology disciplines.

## Data availability statement

The data presented in the study are deposited in the PRIDE repository, accession number PXD040947.

## Ethics statement

The animal study was approved by all animal experiments were carried out in strict accordance with institutional IACUC guidelines, international ARRIVE guidelines, and the principles of the 3Rs of animal research and were approved by BMBWF (Federal Ministry of Education, Science and Research, Austria). All experimental procedures were approved by the BMBWF (GZ 2020–0.592.919). The study was conducted in accordance with the local legislation and institutional requirements.

## Author contributions

DG-V: Conceptualization, Data curation, Formal analysis, Investigation, Methodology, Resources, Supervision, Visualization, Writing – original draft, Writing – review & editing. FX: Data curation, Investigation, Methodology, Resources, Software, Visualization, Writing – review & editing. SG: Data curation, Investigation, Methodology, Writing – review & editing. JS: Methodology, Writing – review & editing. GC: Data curation, Writing – review & editing. MS: Funding acquisition, Supervision, Writing – review & editing.

## Footnotes

^1^ https://www.uniprot.org/proteomes/UP000000589

^2^ https://github.com/vdemichev/diann-rpackage

^3^ https://www.genome.jp/kegg/ko.html

^4^ http://eggnog-mapper.embl.de/

## Supplementary material

Supplementary Figure 1 Identification performance of DIA-NN and MaxQuant software. Precursor identification numbers are plotted against the FDR, estimated using a two-species library method, searching the data against both the microbial protein database (PD2) and the *A. Thaliana* proteome (UP000006548). Each point in the graph corresponds to a decoy (*A. Thaliana*) precursor with the x-axis reflecting its estimated FDR and the respective score threshold and the y-axis representing the number of target microbial precursors at this threshold.

Supplementary Figure 2 Comparisons of peptide identifications. **(A)** Venn diagrams show the unique and overlapped number of identified microbial peptides between the DIA-NN-libE and the DDA-MQ workflows, at increasing amounts of the injected peptides. **(B)** CScore (the score used to calculate precursor q-value in DIANN) distribution (histogram, 30 bins) of peptides uniquely identified in DIA-PASEF workflow (red) and commonly identified in DDA- and DIA-PASEF workflow (gray). **(C)** *Left*: Dynamic range of peptides uniquely identified using DIA-NN-libE (red symbols) or shared with DDA-MQ (grey symbols), when 31.25 ng of peptides were injected. *Right*: Number of detected peptides in each intensity quarter either exclusively identified using DIA-NN-libE (red-bordered boxes) or shared with DDAMQ (grey-bordered boxes).

Supplementary Figure 3 Correlation plots between 3 technical replicates at the lowest peptide quantity tested when using the DDA-MQ (**A**) and the DIA-NN-libE (**B**) workflow.

Supplementary Figure 4 **(A)** Hierarchical classification of annotated taxonomy from Unipept using DIA-NN-libE data. **(B)** Top 30 common GO-MF (Gene Ontology-Molecular Function) enriched in the host proteome (analyzed by Metascape using a cut-off of 3 proteins per function and an adjusted *p*-value < 0.01).

Supplementary Figure 5 Mechanical sensitivity tests of SNI mice **(A)** and SHAM mice **(B)** before (Pre) and after surgery (14D). Each data point on the left side of both graphs corresponds to the ratio of ipsilateral (operated) and contralateral (nonoperated) paw values of individual mice (two-tailed paired t-test; *N* = 4 mice/condition). The right side of both graphs shows the mean (SNI = 0.702, SHAM = 0.031) and individual differences at Pre and 14D with 95% confidence interval.

Supplementary Figure 6 **(A)** Taxonomic annotations in all three conditions at 6 taxa levels. Cutoff: at least 3 unique peptides per taxon (as identified by iMetalab). **(B)** Taxonomic comparison between our study using DIA-PASEF and two published studies using classical DDA. Peptide sequences of two published studies were retrieved from PRIDE using the identifiers and then subjected to iMetalab for taxonomic annotation (cutoff: at least 3 peptides per taxa). In the case of PXD004911 only peptides identified from fecal samples (but not caecal contents) were used for the comparison.

Supplementary Figure 7 Differential expression in the host proteome. **(A)** Shared and unique numbers of regulated host proteins after pairwise comparison (14D versus Pre) in each condition (*p*-value < 0.005). **(B)** The expression patterns of 32 selected host proteins (selected based on *p*-value < 0.005, pair-wise comparison, 14D versus Pre in SHAM) in all three experimental groups. The color code indicates the average ratio (14D/Pre) of each host protein. **(C)** PCA analysis of the protein intensities quantified in the feces of Naive, SHAM, and SNI mice before the surgery.

## Abbreviations

DIA: Data-independent acquisition
DDA: Data-dependent acquisition
PASEF: Parallel accumulation serial fragmentation
FDR: False discovery rate
LC–MS: Liquid chromatography-mass spectrometry
SNI: Spared nerve injury
MBR: Match between runs
MQ: MaxQuant
KEGG: Kyoto Encyclopedia of Genes and Genomes
GO-MF: Gene Ontology Molecular function terms
SIgA: Secreted immunoglobulin A
