# Supplementary figures and images for: Increasing taxonomic and functional characterization of host-microbiome interactions by DIA-PASEF metaproteomics
Source: Front Microbiol. 2023 Oct 16;14:1258703. doi: 10.3389/fmicb.2023.1258703 (PMC10613666; doi:10.3389/fmicb.2023.1258703)

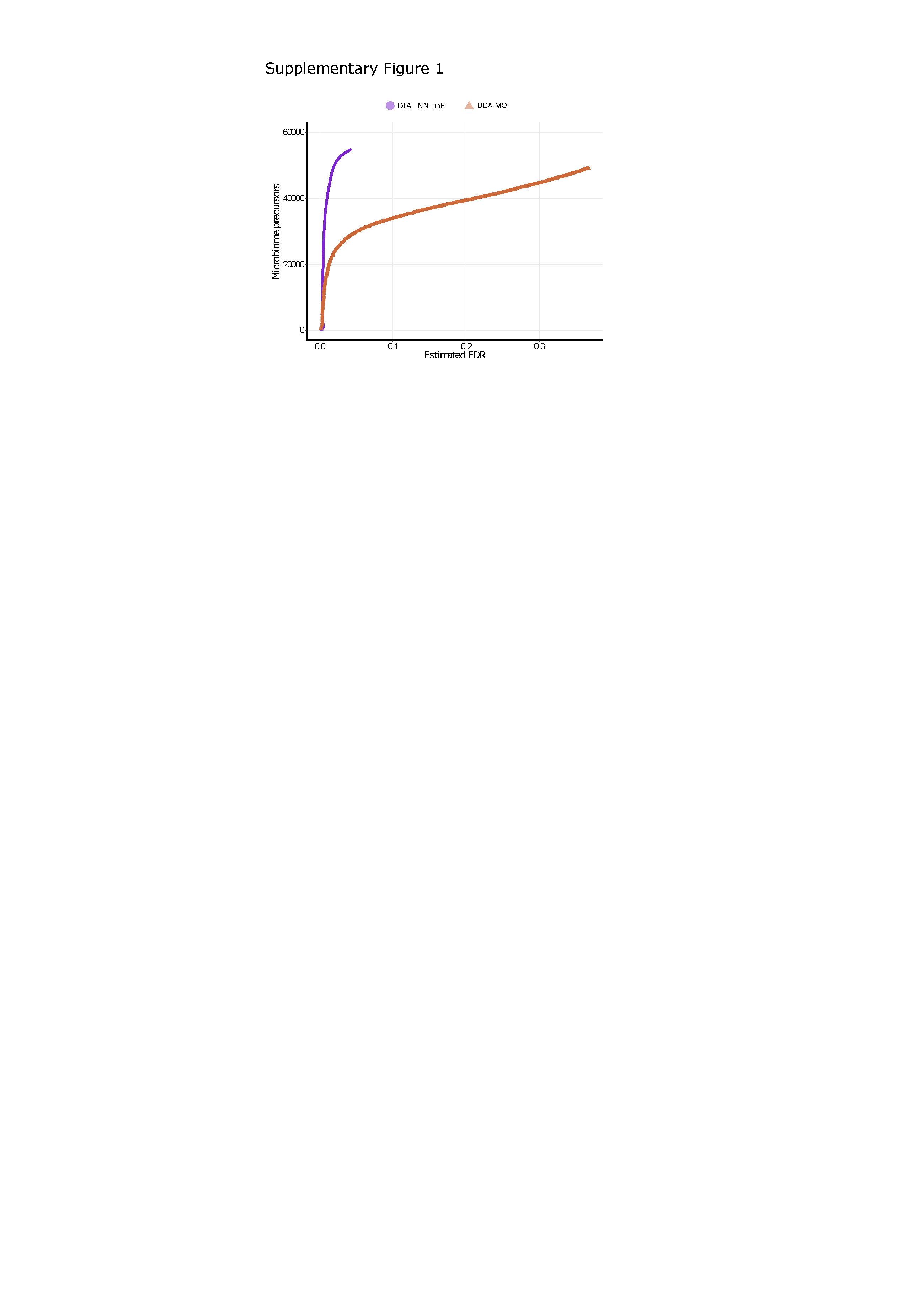

Supplement: Supplementary Figure 1 — Identification performance of DIA-NN and MaxQuant software. Precursor identification numbers are plotted against the FDR, estimated using a two-species library method, searching the data against both the microbial protein database (PD2) and the A. Thaliana proteome (UP000006548). Each point in the graph corresponds to a decoy (A. Thaliana) precursor with the x-axis reflecting its estimated FDR and the respective score threshold and the y-axis representing the number of target microbial precursors at this threshold. [file Image_1.TIFF]

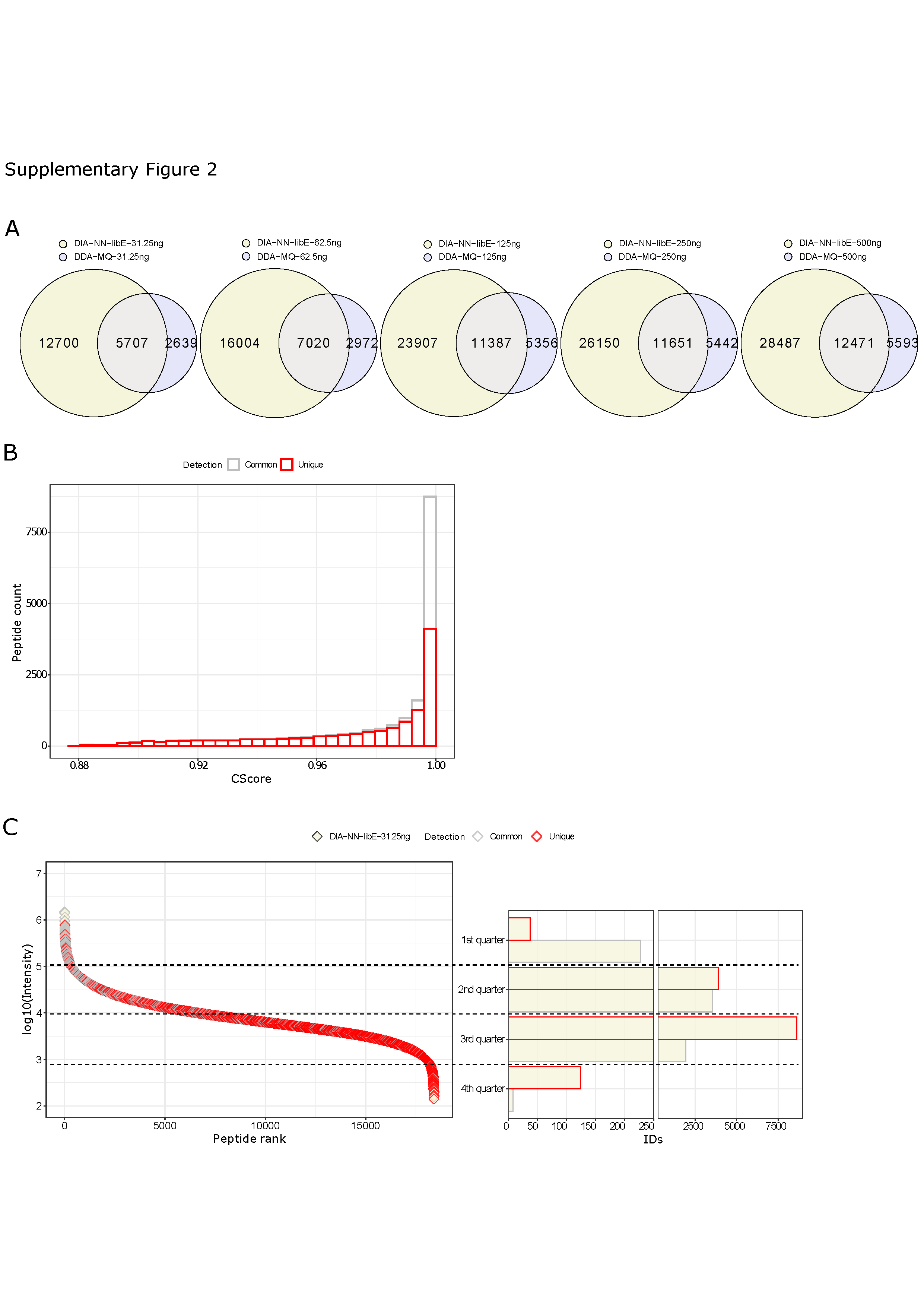

Supplement: Supplementary Figure 2 — Comparisons of peptide identifications. (A) Venn diagrams show the unique and overlapped number of identified microbial peptides between the DIA-NN-libE and the DDA-MQ workflows, at increasing amounts of the injected peptides. (B) CScore (the score used to calculate precursor q-value in DIANN) distribution (histogram, 30 bins) of peptides uniquely identified in DIA-PASEF workflow (red) and commonly identified in DDA- and DIA-PASEF workflow (gray). (C) Left: Dynamic range of peptides uniquely identified using DIA-NN-libE (red symbols) or shared with DDA-MQ (grey symbols), when 31.25 ng of peptides were injected. Right: Number of detected peptides in each intensity quarter either exclusively identified using DIA-NN-libE (red-bordered boxes) or shared with DDAMQ (grey-bordered boxes). [file Image_2.TIFF]

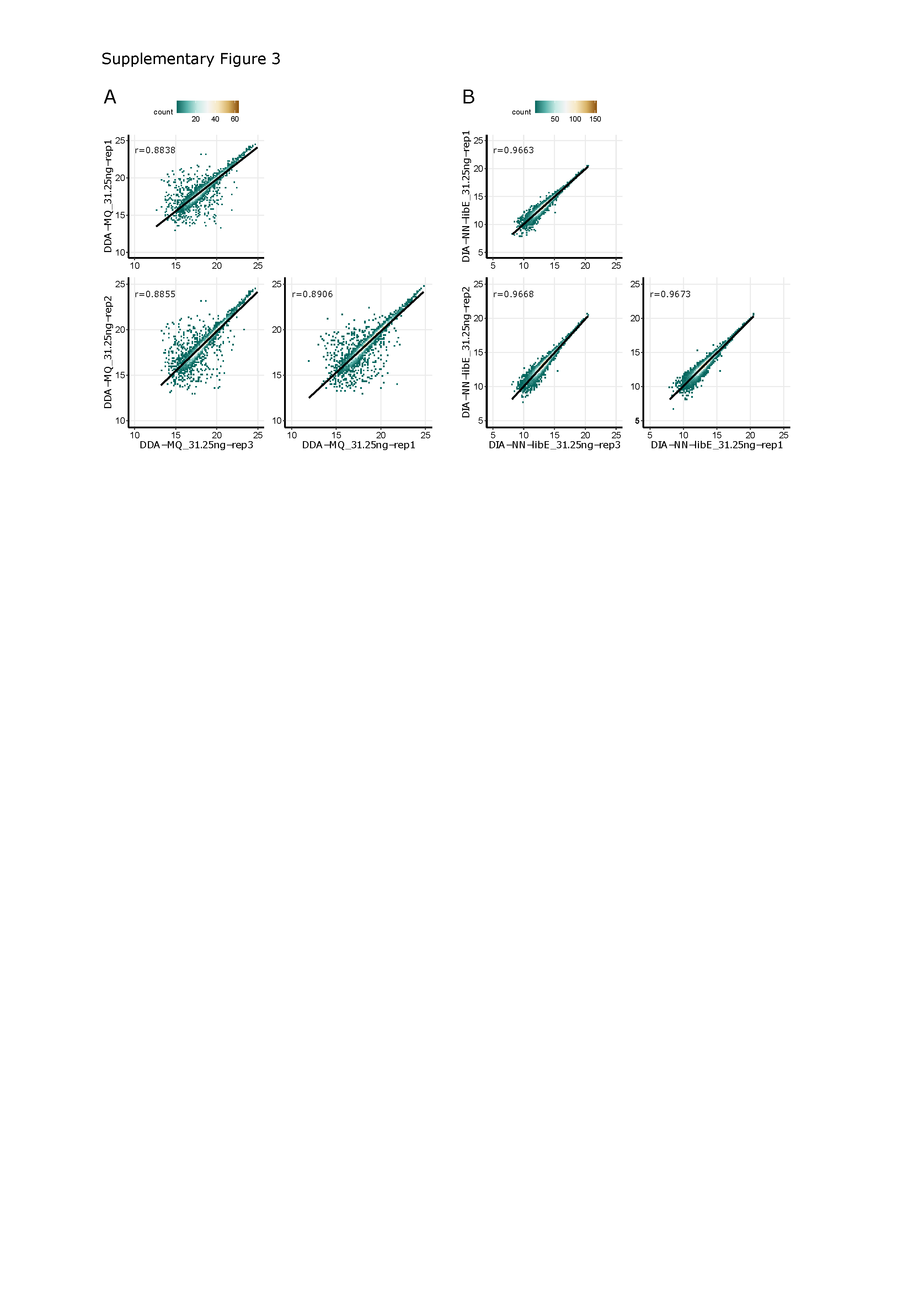

Supplement: Supplementary Figure 3 — Correlation plots between 3 technical replicates at the lowest peptide quantity tested when using the DDA-MQ (A) and the DIA-NN-libE (B) workflow. [file Image_3.TIFF]

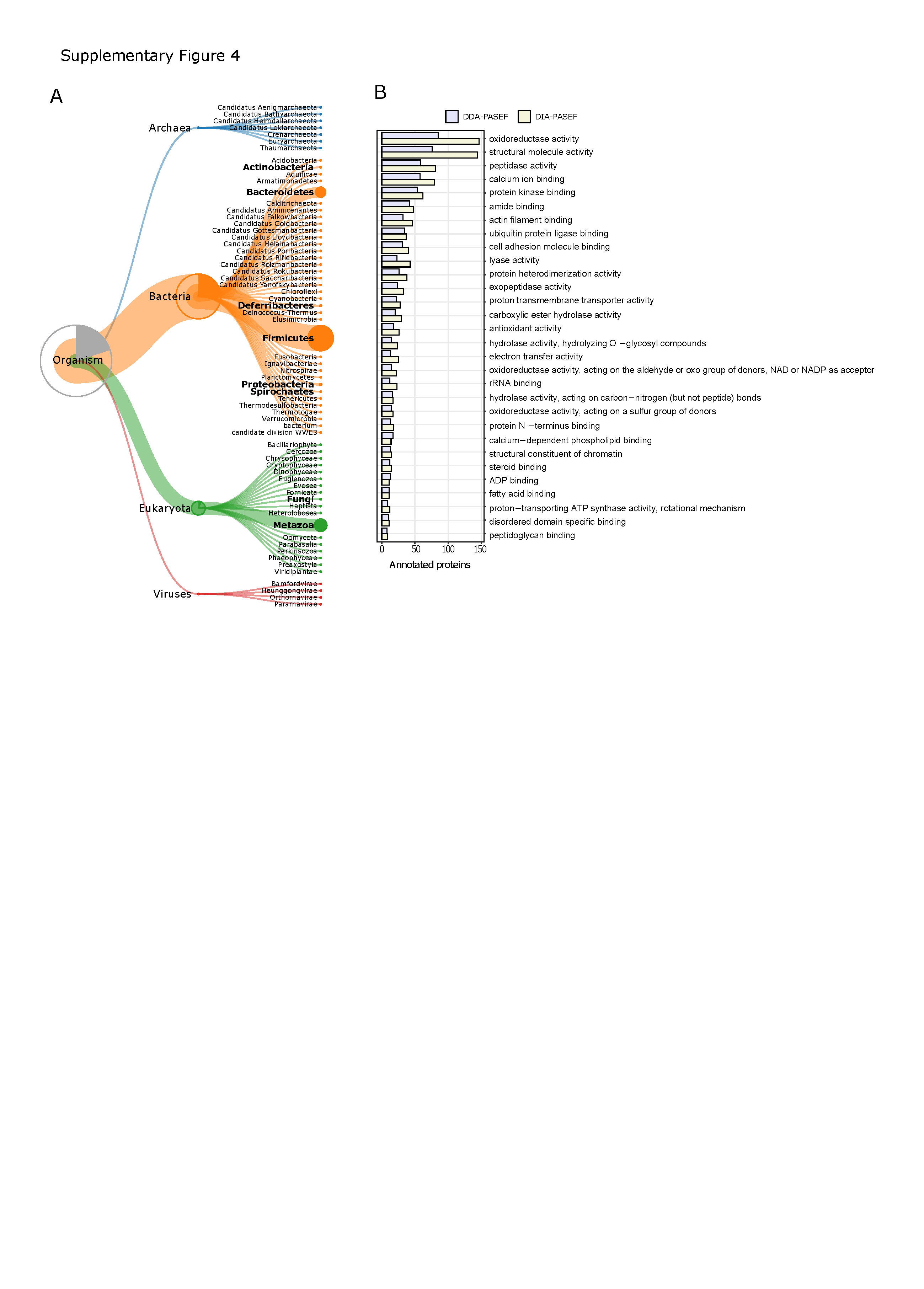

Supplement: Supplementary Figure 4 — (A) Hierarchical classification of annotated taxonomy from Unipept using DIA-NN-libE data. (B) Top 30 common GO-MF (Gene Ontology-Molecular Function) enriched in the host proteome (analyzed by Metascape using a cut-off of 3 proteins per function and an adjusted p-value < 0.01). [file Image_4.TIFF]

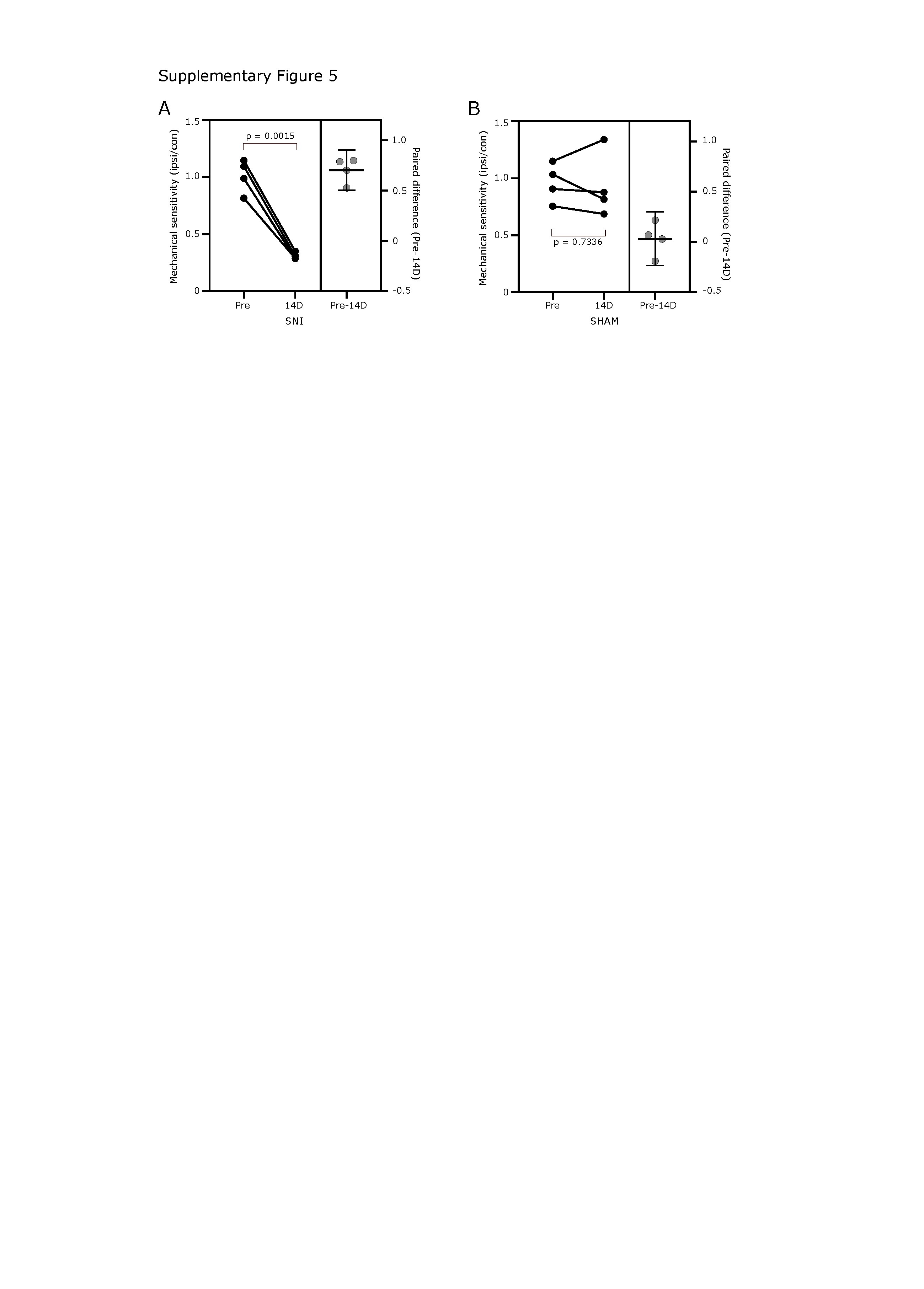

Supplement: Supplementary Figure 5 — Mechanical sensitivity tests of SNI mice (A) and SHAM mice (B) before (Pre) and after surgery (14D). Each data point on the left side of both graphs corresponds to the ratio of ipsilateral (operated) and contralateral (nonoperated) paw values of individual mice (two-tailed paired t-test; N = 4 mice/condition). The right side of both graphs shows the mean (SNI = 0.702, SHAM = 0.031) and individual differences at Pre and 14D with 95% confidence interval. [file Image_5.TIFF]

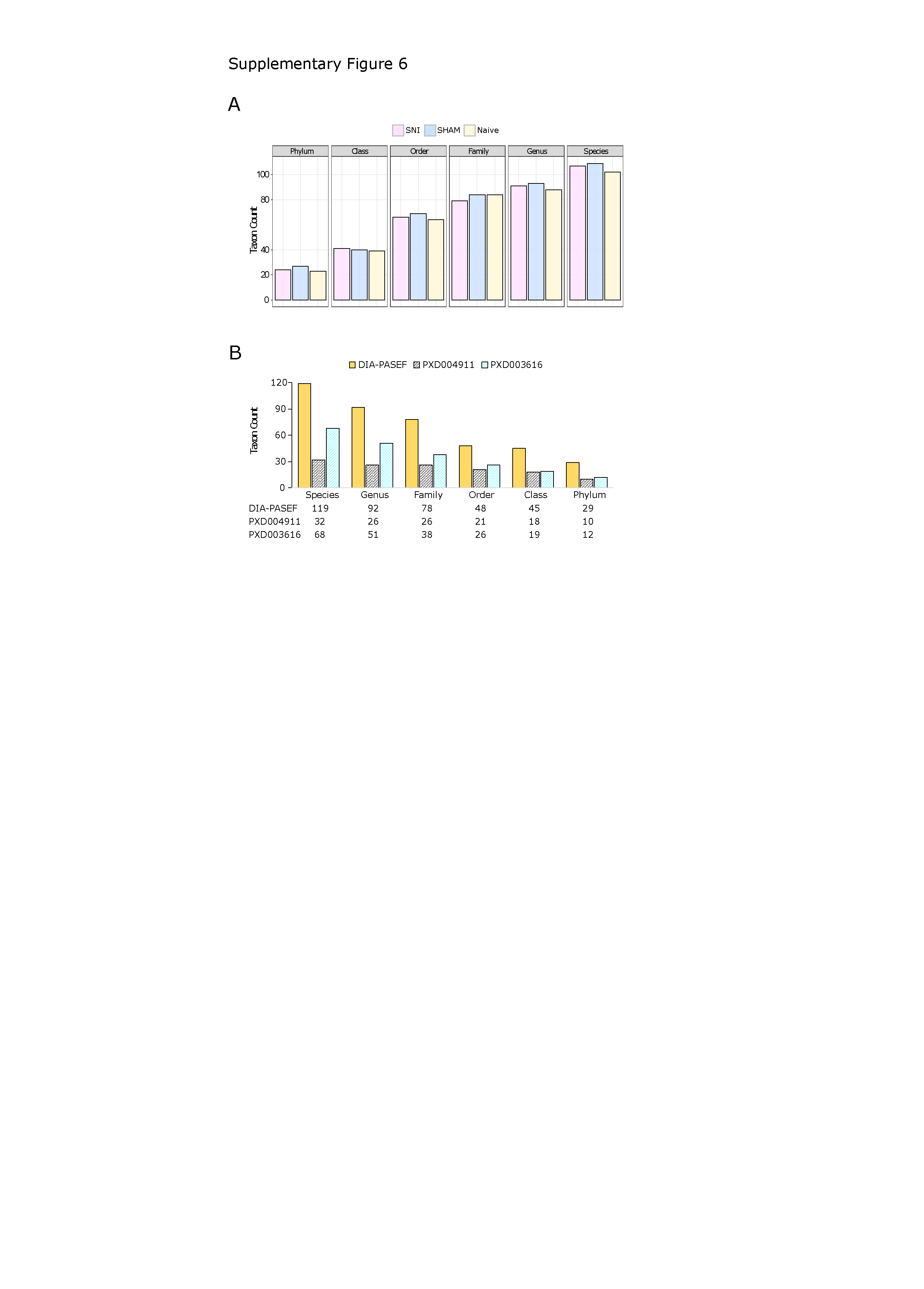

Supplement: Supplementary Figure 6 — (A) Taxonomic annotations in all three conditions at 6 taxa levels. Cutoff: at least 3 unique peptides per taxon (as identified by iMetalab). (B) Taxonomic comparison between our study using DIA-PASEF and two published studies using classical DDA. Peptide sequences of two published studies were retrieved from PRIDE using the identifiers and then subjected to iMetalab for taxonomic annotation (cutoff: at least 3 peptides per taxa). In the case of PXD004911 only peptides identified from fecal samples (but not caecal contents) were used for the comparison. [file Image_6.TIFF]

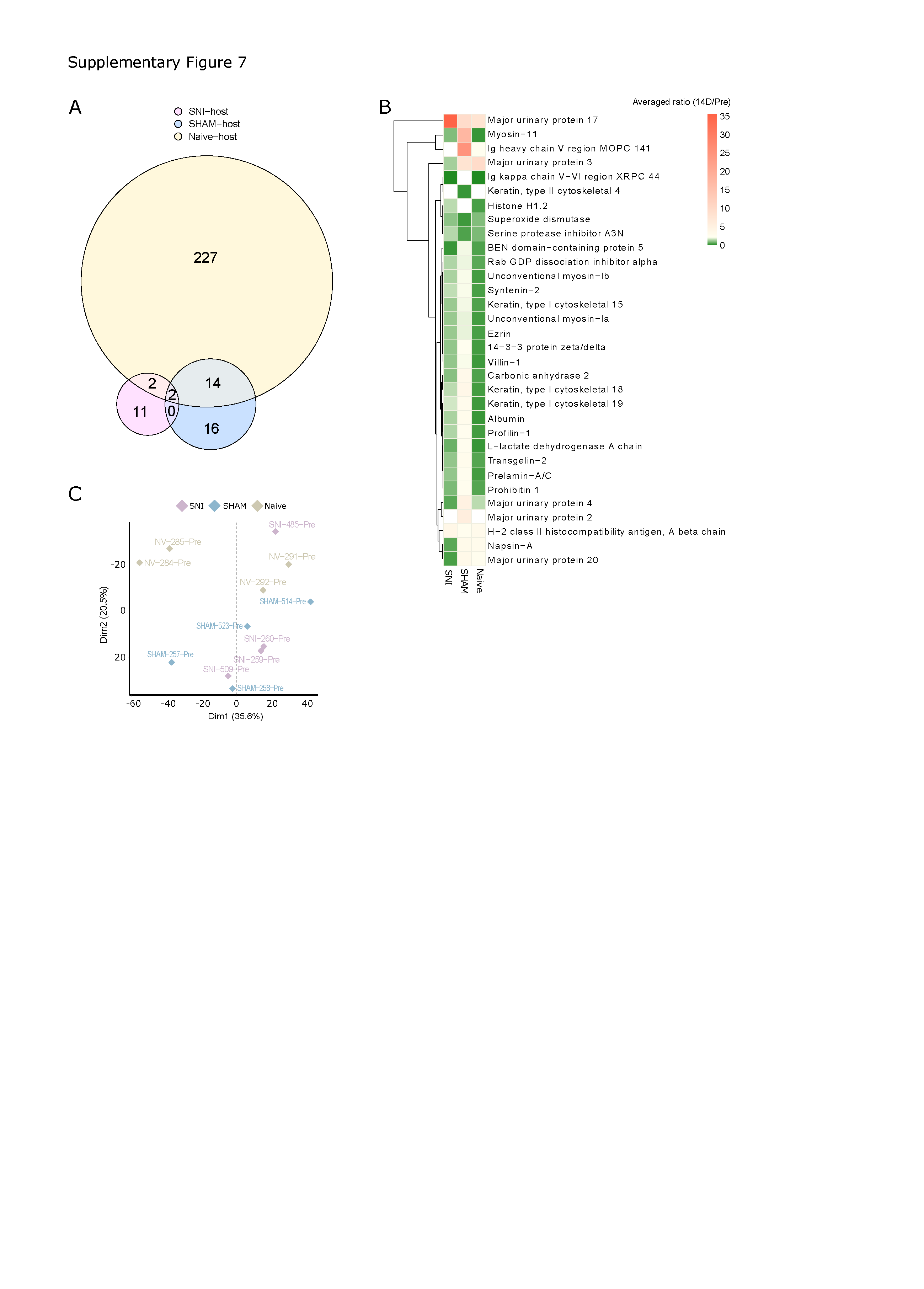

Supplement: Supplementary Figure 7 — Differential expression in the host proteome. (A) Shared and unique numbers of regulated host proteins after pairwise comparison (14D versus Pre) in each condition (p-value < 0.005). (B) The expression patterns of 32 selected host proteins (selected based on p-value < 0.005, pair-wise comparison, 14D versus Pre in SHAM) in all three experimental groups. The color code indicates the average ratio (14D/Pre) of each host protein. (C) PCA analysis of the protein intensities quantified in the feces of Naive, SHAM, and SNI mice before the surgery. [file Image_7.TIFF]
